# Dextromethorphan Exhibits Anti-inflammatory and Immunomodulatory Effects in a Murine Model of Collagen-Induced Arthritis and in Human Rheumatoid Arthritis

**DOI:** 10.1038/s41598-017-11378-8

**Published:** 2017-09-12

**Authors:** Der-Yuan Chen, Chi-Chien Lin, Yi-Ming Chen, Ya-Hsuan Chao, Deng-Ho Yang

**Affiliations:** 1 Division of Allergy, Immunology and Rheumatology, Taichung Veterans General Hospital, Taichung, Taiwan; 2 Institute of Biomedical Science and Rong Hsing Research Center for Translational Medicine, National Chung-Hsing University, Taichung, Taiwan; 3 Faculty of Medicine, National Yang-Ming University, Taipei, Taiwan; 4 Institute of Microbiology and Immunology, Chung-Shan Medical University, Taichung, Taiwan; 5 Department of Biotechnology, Asia University, Taichung, Taiwan; 6 Department of Medical Research, China Medical University Hospital, Taichung, Taiwan; 7 Division of Rheumatology/Immunology/Allergy, Department of Internal Medicine, Taichung Armed Forces General Hospital, Taichung, Taiwan; 8 Department of Medical Laboratory Science and Biotechnology, Central Taiwan University of Science and Technology, Taichung, Taiwan; 9 Division of Rheumatology/Immunology/Allergy, Department of Internal Medicine, Tri-Service General Hospital, National Defense Medical Center, Taipei, Taiwan

## Abstract

Dextromethorphan (d-3-methoxy-17-methylmorphinan, DXM) is a commonly used antitussive with a favorable safety profile. Previous studies have demonstrated that DXM has anti-inflammatory and immunomodulatory properties; however, the effect of DXM in rheumatoid arthritis (RA) remains unknown. Herein, we found that DXM treatment attenuated arthritis severity and proinflammatory cytokine expression levels, including TNF-α, IL-6, and IL-17A, in paw tissues of CIA mice. DXM treatment also reduced serum TNF-α, IL-6, and IL-17A levels of CIA mice and patients with RA. DXM further decreased the production of anti-CII IgG, IFN-γ, and IL-17A in collagen-reactive CD4^+^ T cells extracted from the lymph nodes of CIA mice. *In vitro* incubation of bone marrow–derived dendritic cells with DXM limited CD4^+^ T-cell proliferation and inflammatory cytokine secretion. In conclusion, our results showed that DXM attenuated arthritis symptoms in CIA mice and significantly reduced proinflammatory cytokines in patients with RA, suggesting that it can be used as an anti-arthritic agent.

## Introduction

Rheumatoid arthritis (RA) is a chronic inflammatory disease is characterized by the infiltration of macrophages, B cells and T cells and by synovial hyperplasia and bone erosions^1^. Macrophage-derived proinflammatory cytokines such as tumor necrosis factor (TNF)-α and interleukin (IL)-6 are crucial mediators of rheumatoid synovitis and subsequent bone destruction^2, 3^. IL-17A can amplify the inflammatory cascade^4^, and enhanced expression of IL-17 has been observed in rheumatoid synovium^5^. Increasing evidence has shown that alterations in proinflammatory cytokines are both a possibility an important pathogenic factor and a potential target for therapeutic intervention in RA^6–10^.

Based on the 2013 European League Against Rheumatism (EULAR) recommendations for the management of RA^11^, methotrexate (MTX) alone or in combination with conventional synthetic disease-modifying antirheumatic drugs (csDMARDs) such as sulfasalazine, hydroxychloroquine, and leflunomide should be part of the initial therapeutic strategy for patients with active RA. If a treatment target is not reached, biologic DMARDs (bDMARDs) should be considered if poor prognostic factors are present^12, 13^. In consideration of the high cost of bDMARDs and increased infection rate of patients administrated these biologic agents, add-on therapeutic agents targeting immune or inflammatory responses are necessary in RA.

Dextromethorphan (d-3-methoxy-17-methylmorphinan, DXM), a dextrorotatory morphinan, is a widely used and central acting antitussive with a proven safety profile^14^. In addition to its antitussive effect, DXM has also been shown to have anti-inflammatory and immunomodulatory properties. For example, DXM reduced the production of pro-inflammatory factors (such as TNF-α) from activated microglia or macrophages in the brain and aortic sinuses^15–17^ as well as the levels of group A streptococcal (GAS)-induced pro-inflammatory cytokines and chemokines in a mouse model^18^. Also, DXM has been shown that it could protect mice from lipopolysaccharide (LPS)/GalN-induced endotoxin shock and liver injury through its anti-inflammatory effects^19^. Moreover, a precious study demonstrated that DXM was able to attenuate oxidative stress and inflammatory markers in habitual smokers^20^. Our recent studies further indicated that DXM could inhibit the activation and function of mouse bone marrow–derived dendritic cells (BMDCs) and human monocyte–derived dendritic cells (MDDCs)^21^. DXM also decreased LPS-induced secretion of TNF-α, IL-6 and IL-12, which drives the Th1 response^21^. Although a few studies have addressed the anti-inflammatory and immunomodulatory potential of DXM, the ability of DXM ability to protect against arthritis, as well as the clinical implications of DXM in patients with RA, remains unknown. The aims of the present study were as follows: (1) to evaluate the therapeutic effects of DXM in a murine RA model (collagen-induced arthritis, CIA) and in patients with RA, and (2) to explore the possible molecular mechanisms underlying the therapeutic effects of DXM.

## Results

### DXM treatment ameliorated mouse CIA

**Figure 1:**
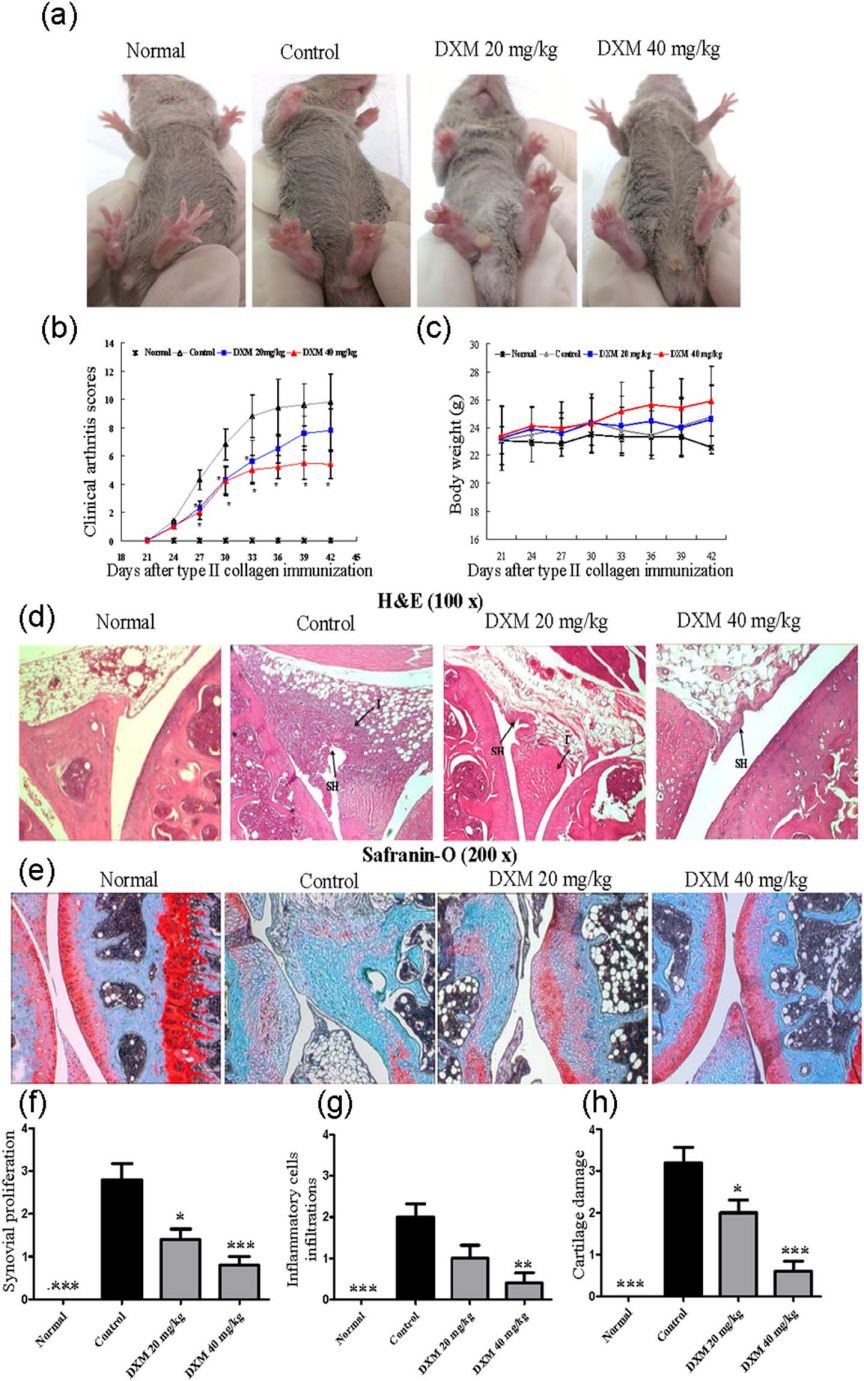
Therapeutic effects of dextromethorphan (DXM) on articular inflammation in mice with collagen-induced arthritis (CIA). DBA/1 mice with CIA were orally treated with DXM at 20 or 40 mg/kg daily from day 21 to day 42. Representative photographs of the hind paws of CIA mice on day 42 were shown in (**a**). Clinical arthritis scores (**b**) and the body weight changes (**c**) were determined on indicated day after immunization. The ankle joint sections were stained with H&E (**d**) and Safranin O (**e**). The comparisons in synovial proliferation (**f**), the inflammatory cell infiltrations (**g**), and cartilage damage (**h**) between normal mice, CIA/PBS group (as control group), CIA/DXM 20 mg/kg group, and CIA/DXM 40 mg/day group. Values in **b**, **c**, **f**, **g**, and **h** are the mean ± SEM of 6 mice per group; results are representative of 3 independent experiments. *p < 0.05, **p < 0.01, ***p < 0.001, versus CIA/PBS group, as determined by a one-way ANOVA with Tukey’s multiple comparison test.

Mouse CIA has been widely utilized to study arthritic diseases that have many pathological features similar to those of human RA^22^. First, we investigated the possible therapeutic effect of DXM on a CIA DBA/1 mouse model. DXM was orally administered to mice at 20 or 40 mg/kg daily from days 21–42 after collagen immunization. As shown in Fig. 1a,b, compared with the PBS-treated group, DXM (20 or 40 mg/kg) significantly ameliorated severe swelling, erythema, and joint rigidity in the hind paws confirmed by visual inspection (Fig. 1a) and arthritis scores (Fig. 1b). There were no differences in bodyweight changes between the groups (Fig. 1c). To further evaluate the histological changes in the ankle joints, the mice were sacrificed at the end of experiments (day 42), and their joints were stained with H&E. The joints of mice treated with 20 or 40 mg/kg of DXM daily demonstrated less inflammatory cell infiltration and synovial hyperplasia (Fig. 1d,f,g). In addition, selected joint sections were stained with Safranin O to evaluate proteoglycan content in the articular cartilage. The CIA group had reduced Safranin-O staining (Fig. 1e), indicating diminished proteoglycan content. In contrast, in the CIA animals administered DXM treatments, the decrease in proteoglycan staining was less, as shown by histological grading of Safranin-O staining. These results suggest that DXM has the potential to ameliorate the development of CIA.

### DXM suppressed inflammatory responses in the joints of mice with CIA

**Figure 2:**
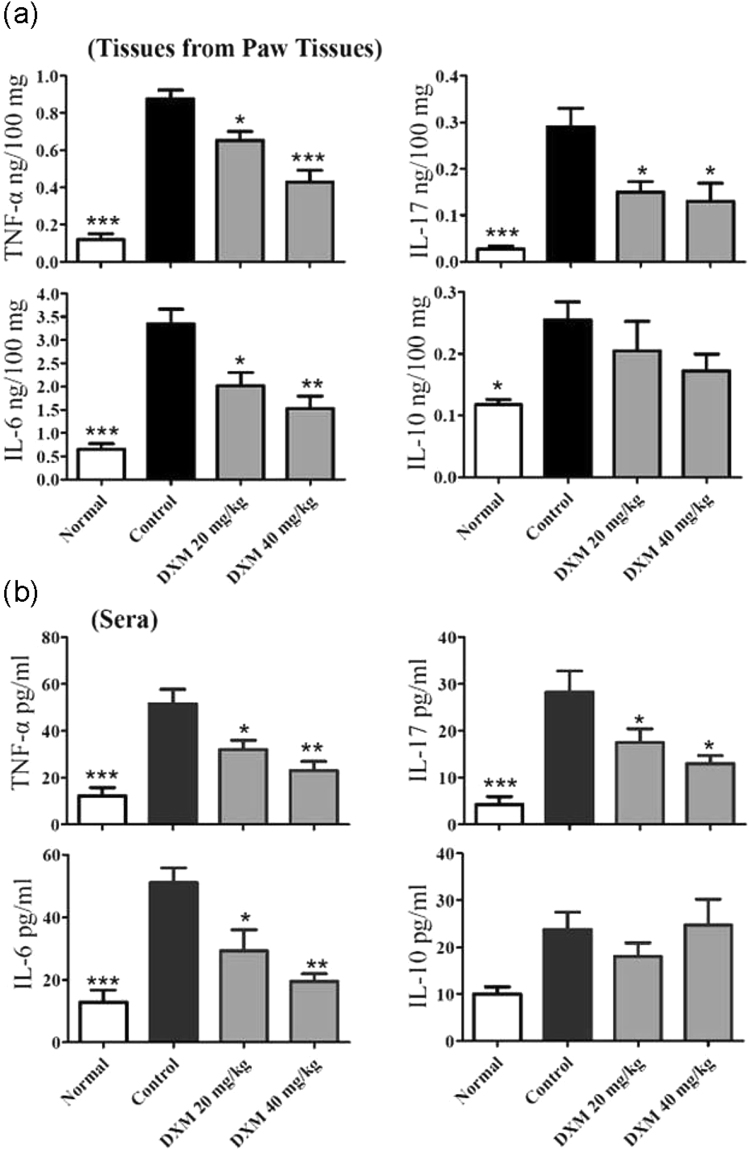
DXM selectively inhibited the production of cytokines in mice with CIA. Mice with CIA were untreated or treated with DXM and data were representative from three experiments. The cytokine levels in (**a**) paw tissues and (**b**) sera were determined by ELISA. Bar graphs represent the mean ± SEM with six mice per group pooled from two independent experiments. *p < 0.05, **p < 0.01, ***p < 0.001, versus CIA/PBS group, as determined by a one-way ANOVA with Tukey’s multiple comparison test.

We investigated the mechanisms underlying the decreased occurrence and severity of CIA following DXM treatment. Levels of proinflammatory cytokines including TNF-α, IL-6, and IL-17A and anti-inflammatory cytokine IL-10 in the paw tissues and sera collected at the end of the experiments (day 42) were measured using ELISA. These cytokines are involved in the development of CIA. The levels of TNF-α, IL-6, IL-17A, and IL-10 in the paws of mice with CIA were significantly higher than those of control mice. Compared with the mice with CIA, lower TNF-α, IL-6, and IL-17A levels were significantly observed in the paw tissues of DXM-treated mice (Fig. 2a). The mice administered DXM also had significantly lower serum TNF-α, IL-6, and IL-17A levels, as compared with the mice with CIA (Fig. 2b). However, there was no difference in IL-10 levels between the DXM-treated CIA group and untreated group. These data suggest that the administration of DXM may have a therapeutic effect on CIA severity by inhibiting the local and systemic production of proinflammatory cytokines.

### DXM decreased autoAb levels in serum and suppressed T-cell expansion in LNs

**Figure 3:**
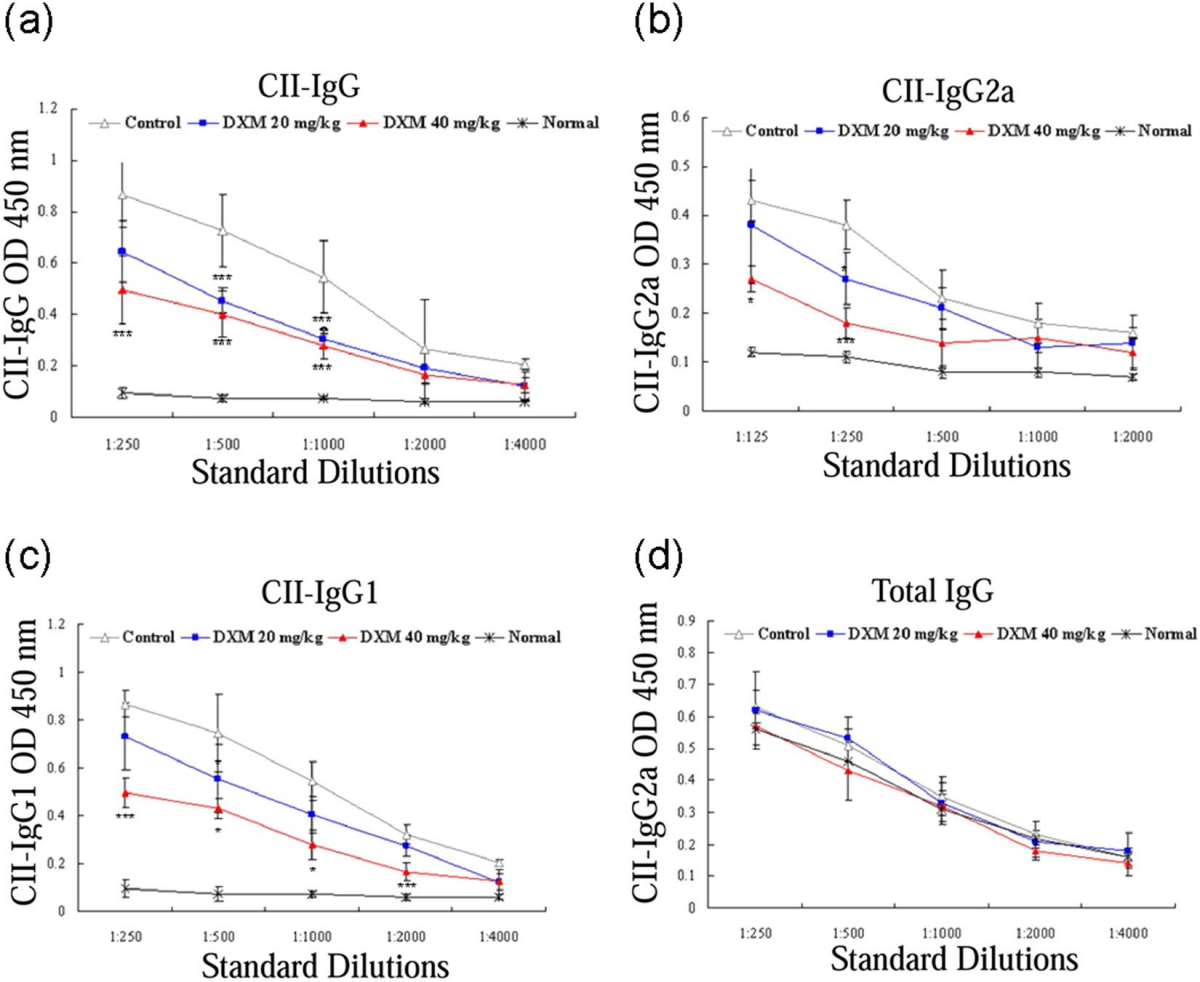
DXM inhibited autoantibody production in mice with CIA. The antibody levels of mice in serum were determined by ELISA. DXM treatment significantly inhibited the production of CII-specific IgG (**a**), IgG2a (**b**), and IgG1 (**c**), but not total IgG (**d**) in mice with CIA. Bar graphs represent the mean ± SEM with six mice per group pooled from two independent experiments. *p < 0.05, and ***p < 0.001, versus CIA/PBS (as control group), as determined by a one-way ANOVA with Tukey’s multiple comparison test.

Both humoral and cellular immune responses to CII play an important role in CIA^22, 23^. Our results showed a significant increment of CII-specific IgG, IgG1, and IgG2a levels in serum collected at day 42 in all CIA mice, as compared with control mice (Fig. 3a–c). Levels of serum CII-specific autoAb were significantly lower in CIA mice treated with 20 or 40 mg/kg of DXM, as compared with CIA mice. However, DXM treatment had no significant effect on total IgG levels (Fig. 3d), suggesting that DXM selectively affects collagen-specific B-cell responses in CIA rather than acting as a general B-cell–suppressing factor.

**Figure 4:**
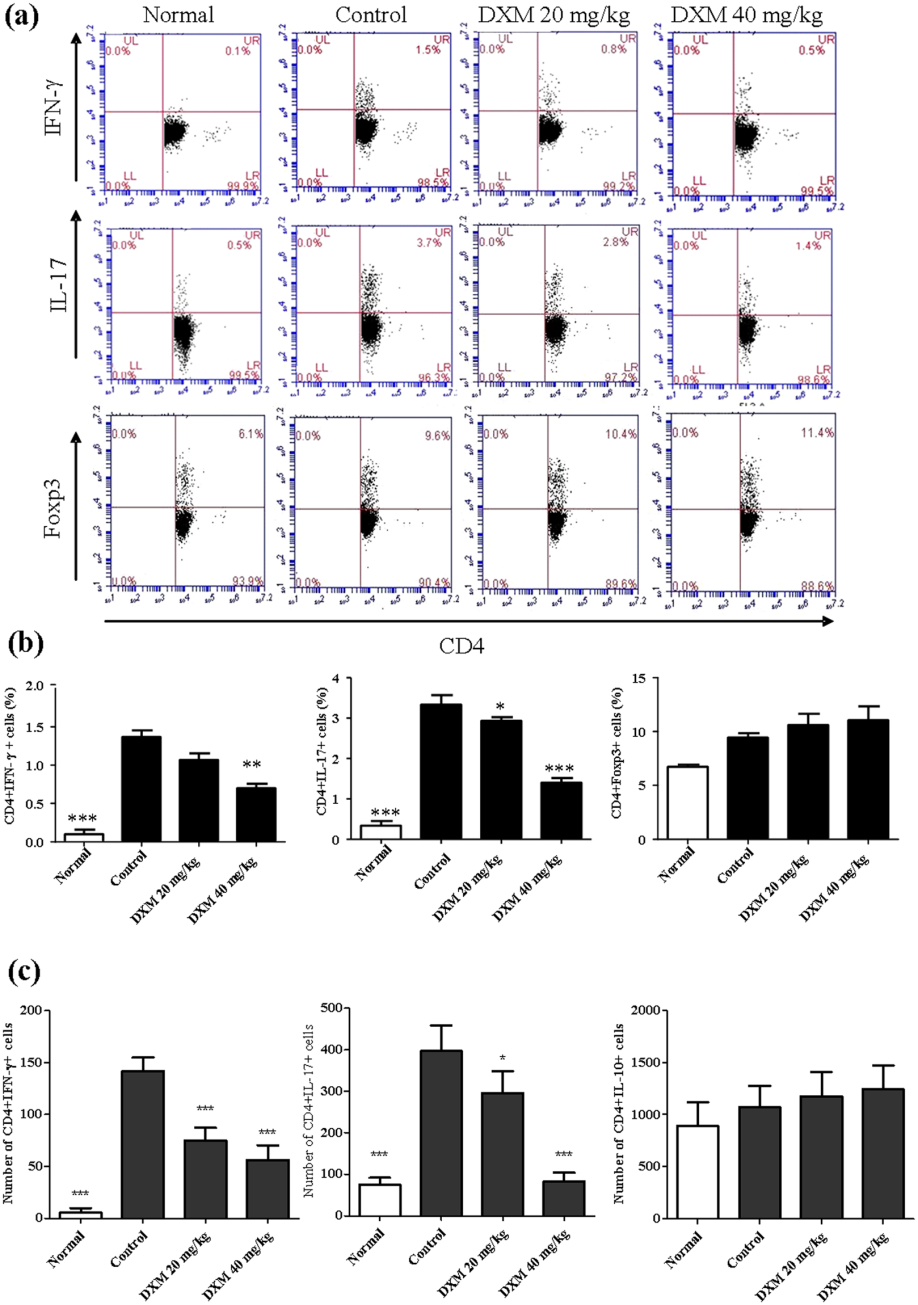
DXM treatment suppressed T-cell expansion and inflammatory cytokine production *in vivo*. CIA mice were untreated or treated with DXM, and inguinal LNs were collected and analyzed. The inguinal LN cells (2 × 10^6^/mL) were cultured with chicken CII (20 μg/mL) for 72 h and then stained with anti-CD4 and anti- IFN-γ, anti-IL-17A, or anti-Foxp3 Abs and analyzed by flow cytometry. Flow cytometric dot-plots (**a**) of intracellular staining of IFN-γ, IL-17A, and Foxp3 in T cells were obtained from inguinal LN cells of one representative mouse from each group. The comparison in the percentages of IFN-γ-, IL-17A-, and Foxp3 positive-CD4^+^ T cells between four groups was shown in (**b**). Bar graphs represent the mean ± SEM with six mice per group pooled from two independent experiments. **(c)** the absolute number of IFN-γ-, IL-17A-, and Foxp3 positive-CD4^+^ T cells in LNs were also recorded. *p < 0.05, **p < 0.01, ***p < 0.001, versus CIA/PBS group, as determined by a one-way ANOVA with Tukey’s multiple comparison test.

To examine the effect of DXM on T-cell responses, inguinal LN cells from CIA mice treated with 20 or 40 mg/kg of DXM and untreated CIA mice were stimulated *ex vivo* with CII for 72 h. After stimulation with CII, a flow cytometric analysis showed that CD4^+^ T cells derived from DXM-treated CIA mice had a significantly lower production of Th1 and Th17 cytokines, as compared with CD4^+^ T cells from untreated CIA mice. However, there was no difference in the population of FOXP3^+^CD4^+^ regulatory T cells between the two groups (Fig. 4a,b). Consistent with the reduced frequency of Th1 and Th17 cells, the absolute number of both T-cell subsets was also markedly decreased but did not affect Treg cells in LNs from CIA mice with DXM treatment (Fig. 4c). Taken together, these findings, suggest that the reduction in Th1 and Th17 was not caused by an increase in Treg cells.

### DXM decreased the capacity of DCs to stimulate CII-specific T-cell activation *in vitro*

**Figure 5:**
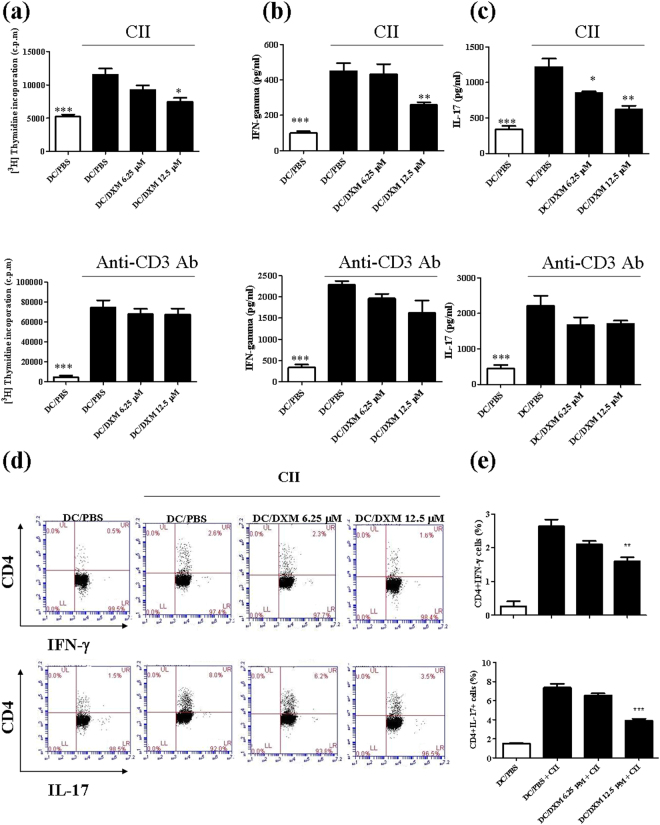
DXM treatment inhibited CII-specific T cell activation induced by CII-stimulated DCs. BMDCs from DBA/1 mice were incubated with DXM (6.25 µM or 12.5 µM) or PBS (as control) for 24 h. CD4^+^ T cells from inguinal LN cells of CIA mice on day 35 were added to BMDCs culture, followed by stimulation with chicken CII or anti-CD3 Ab for 96 h. T cell proliferation (**a**) induced by CII stimulation or anti-CD3 Ab was determined by [^3^H]-thymidine incorporation for 18 h before the end of the incubation. The levels of IFN-γ (**b**) and IL-17A (**c**) in supernatants were determined by ELISA, and the percentages (**d**) of IFN-γ- or IL-17A-producing CII-specific CD4^+^ T cells were determined using flow cytometry. The dot plot data were from one representative sample. The comparison in the percentages of IFN-γ- or IL-17A-positive CD4^+^ cells between four groups was shown in (**e**). Bar graphs are mean ± SEM from triplicate cultures; results are representative of three independent experiments. *p < 0.05, **p < 0.01, ***p < 0.001, versus DC/PBS group, as determined by a one-way ANOVA with Tukey’s multiple comparison test.

Our previous study showed that DXM inhibited LPS-induced maturation of mouse and human DC and suppressed the capacity of DC to stimulate proliferation of and IFN-γ production by syngeneic T cells^21^. Thus, to further elucidate the cellular mechanism of DXM treatment on CIA, we investigated the effects of DXM on the capacity of DCs to activate CII-specific T cells. For this study, We first validated our *in vitro* system by treating BMDC cells with CII antigen. As expected, these CII-treated BMDCs exhibited higher T-cell proliferation (Fig. 5a) and the production of IFN-γ and IL-17A compared to untreated BMDCs (Fig. 5b,c). Then, we pretreated BMDCs with DXM overnight prior to administration of the CII antigen, anti-CD3 antibody, or medium stimulation for next 72 hrs. The results in Fig. 5 indicate that the CII-induced cell proliferation and cytokine production as well as the frequencies of IFN-γ–producing and IL-17–producing CD4 T cells could be significantly reduced by DXM pretreatment in a dose-dependent fashion. Of note, the DXM treatment did not significantly alter the anti-CD3 Ab-stimulated cellular proliferative responses or cytokine productions by T lymphocytes *in vitro*.

**Figure 6:**
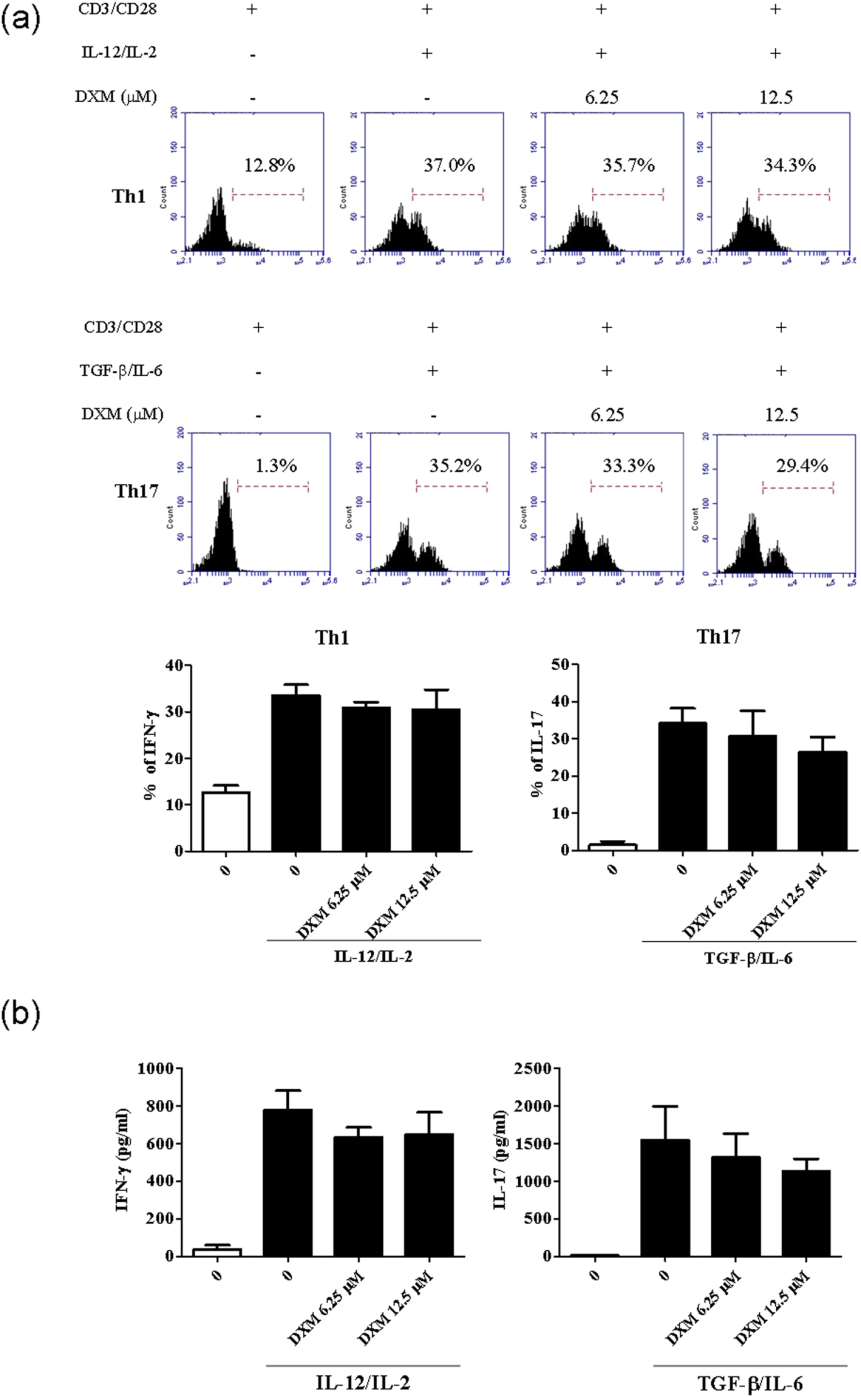
DXM did not regulates Th1 and Th17 expansion *in vitro*.Th1 and Th17 cells were expanded from DBA/1 mouse CD4+ T cells *in vitro* in the presence of cytokines and treated with DXM. (**a**) The frequency of Th1 and Th17 cells were examined by flow cytometry. (**b**) The production of IFN-g and IL-17 cytokine in culture supernatant were determined by ELISA assay. *p < 0.05, **p < 0.01, ***p < 0.001, versus PBS treated group, as determined by a one-way ANOVA with Tukey’s multiple comparison test.

In order to clarify whether or not the DXM could directly affect T cell-linage differentiation, We established an *in vitro* culture system where allowed us to expand murine Th1 and Th17 cell populations by administrating either IL-2 and IL-12 for Th1 differentiation or TGF-β and IL-6 cytokines for Th17 differentiation in the presence or absence of the DXM treatment. As shown in Fig. 6, DXM treatment on naïve T cells did not significantly alter the proliferating frequencies toward to Th1 or Th17 (Fig. 6a), and DXM did not profoundly modulate the production of IFN-γ and IL-17 in cellular supernatant (Fig. 6b) in polarized Th1 and Th17 differentiation conditions. These results suggest that DXM may suppress antigen-specific T-cell responses by working on BMDCs.

### Baseline clinical characteristics of patients with RA

As illustrated in Table 1, the majority of RA patients were female, and all patients had active disease (DAS28, mean ± SD, 4.22 ± 0.54) before starting add-on DXM therapy. There were no significant differences in baseline demographic data, clinical characteristics, RF or anti-CCP Ab positivity, DAS28, CRP levels, daily corticosteroid dose, weekly methotrexate dose, proportion of DMARDs used, or proportion of comorbidities between patients with and without DXM add-on therapy.

**Table 1:** Demographic data and laboratory findings of rheumatoid arthritis patients with or without add-on dextromethorphan (DXM) therapy.

	With DXM (n = 24)	Without DXM (n = 24)
Mean age at entry, years	52.2 ± 11.6	52.8 ± 10.5
Female proportion	19 (79.2%)	19 (79.2%)
Disease duration, years	9.5 ± 2.8	8.5 ± 2.8
RF positivity	21 (87.5%)	20 (83.3%)
Anti-CCP positivity	18 (75.0%)	17 (70.9%)
Baseline CRP, mg/dl	1.7 ± 1.1	1.5 ± 1.0
Baseline DAS28	4.23 ± 0.58	4.21 ± 0.52
Daily steroid dose, mg/day	6.3 ± 2.1	6.0 ± 2.1
DMARDs used		
Methotrexate, weekly dose, mg	12.2 ± 2.7	12.1 ± 2.2
Sulfasalazine	20 (83.3%)	19 (79.2%)
Hydroxychloroquine	18 (75.0%)	19 (79.2%)
Leflunomide	10 (41.7%)	8 (33.3%)
Cyclosporine	8 (33.3%)	7 (29.2%)
Comorbidities		
Diabetes mellitus	2 (8.3%)	1 (4.2%)
Hypertension	4 (16.7%)	5 (20.8%)
Cardiovascular disease	1 (4.2%)	0 (0.0%)

^#^Data are presented as mean ± SD or number (percentage).

RF: rheumatoid factor; Anti-CCP: anti-cyclic citrullinated peptide antibodies; CRP: C-reactive protein; DAS28: disease activity score for 28-joints; DMARDs: disease-modifying anti-rheumatic drugs.

### Comparison of EULAR response in patients with RA who had or did not have add-on DXM therapy

**Figure 7:**
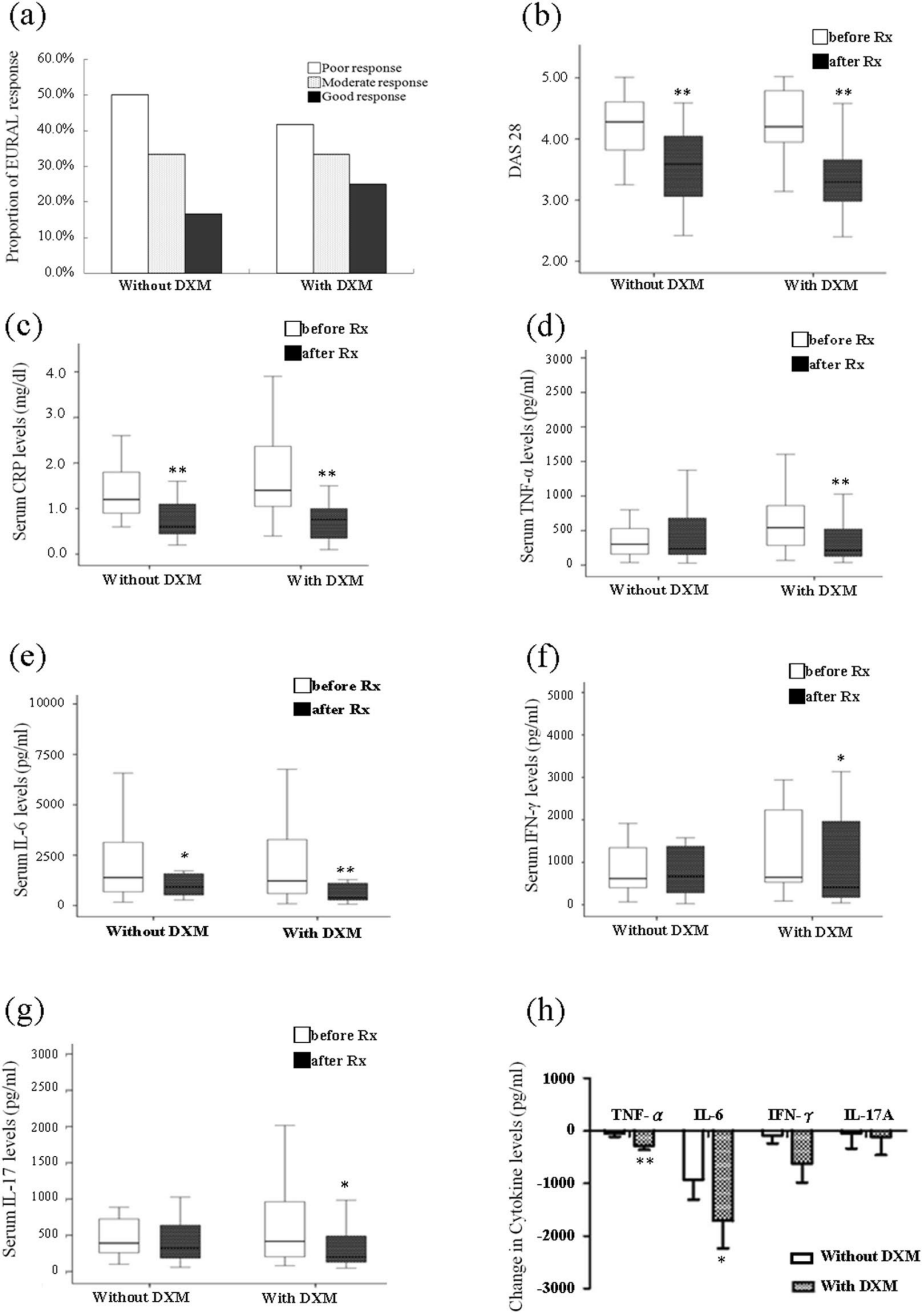
Comparison of RA patients with or without add-on DXM therapy. (**a**) The comparison of EULAR therapeutic response assessed at the 6^th^ month between RA patients with or without add-on DXM therapy was determined by Fisher’s exact test. The changes in disease activity parameters including (**b**) DAS28, (**c**) CRP levels, the levels of proinflammatory cytokines in serum including (**d**) TNF-α, (**e**) IL-6, (**f**) IFN-γ, and (**g**) IL-17A after 6-month therapy with add-on DXM in RA patients. The data were presented as box-plot diagrams, with the box encompassing the 25^th^ percentile (lower bar) to the 75^th^ percentile (upper bar), and the horizontal line within the box indicated the median value. (**h**) The comparison of the decrement of TNF-α, IL-6, IFN-γ and IL-17A levels between patients with or without add-on DXM therapy. Data were presented as mean ± SEM. *p < 0.05, **p < 0.005, versus before treatment, determined by the Wilcoxon signed rank test.

Compared with patients without add-on DXM therapy, patients who received 6-month add-on DXM therapy had a slightly higher rate of good and total EULAR responses (25.0% versus 16.7%, respectively, for good response and 58.3% versus 50.0%%, respectively, for total EULAR response) (Fig. 7a).

### Change in RA activity parameters after 6-month therapy withadd-on DXM

As shown in Fig. 7b,c, DAS28 and CRP levels were significantly decreased in patients with 6-month add-on DXM therapy (median = 4.20, interquartile range [IQR] 3.94–4.80 versus 3.30, IQR 2.98–3.69 and median = 1.40 mg/dl, IQR 1.03–2.48 mg/dl versus 0.75 mg/dl, IQR 0.33–1.05 mg/dl, respectively; both p < 0.001), as compared with patients who did not have add-on DXM therapy (median = 4.28, IQR 3.79–4.61 versus 3.59, IQR 2.99–4.06 and median = 1.20 mg/dl, IQR 0.90–1.80 mg/dl versus 0.60 mg/dl, IQR 0.42–1.15 mg/dl, respectively; both p < 0.001).

### Change in proinflammatory cytokines after 6-month therapy withadd-on DXM

After add-on DXM therapy for 6 months, significantly decreased levels of TNF-α, IL-6, IFN-γ, and IL-17A (median = 541.9 pg/ml, IQR 286.5–863.5 pg/ml versus 214.1 pg/ml, IQR 118.7–568.1 pg/ml, respectively, p < 0.005; median = 1222.7 pg/ml, IQR 598.1–3343.2 pg/ml versus 386.3 pg/ml, IQR 255.8–1137.3 pg/ml, respectively, p < 0.005; median = 646.4 pg/ml, IQR 527.8-2499.8 pg/ml versus 406.6 pg/ml, IQR 155.4–2085.9 pg/ml, respectively, p < 0.05; and median = 416.4 pg/ml, IQR 201.1–1041.4 pg/ml versus 203.2 pg/ml, IQR 121.0-564.4 pg/ml, %, respectively, p < 0.05) were observed. For patients without add-on DXM therapy, significantly decreased IL-6 levels (median = 1388.4 pg/ml, IQR 681.9–3539.8 pg/ml versus 925.5 pg/ml, IQR 519.6–1647.7 pg/ml, p < 0.05, respectively, Fig. 7e) were observed. However, there was no significant change in TNF-α levels (median = 302.8 pg/ml, IQR 151.0–581.4 pg/ml versus 236.0 pg/ml, IQR 151.2–707.7 pg/ml, respectively, Fig. 7d), IFN-γ (median = 617.4 pg/ml, IQR 402.9–1402.9 pg/ml versus 672.2 pg/ml, IQR 250.3–1478.1 pg/ml, respectively, Fig. 7f), or IL-17A (median = 391.4 pg/ml, IQR 248.5–760.8 pg/ml versus 320.0 pg/ml, IQR 157.8–663.9 pg/ml, respectively, Fig. 7g) for patients who were not administered add-on DXM therapy.

As shown in Fig. 7h, a significant decrease in TNF-α and IL-6 levels was observed in patients with add-on DXM therapy (310.5 ± 72.5 pg/ml and 1763.2 ± 516.5 pg/ml, respectively), as compared with patients without DXM therapy (49.1 ± 61.9 pg/ml, p < 0.005 and 923.8 ± 384.4 pg/ml, respectively p < 0.05). Although there was no statistical significance, patients administered DXM therapy experienced a greater decrease in IFN-γ and IL-17A levels (622.4 ± 359.2 pg/ml and 124.8 ± 347.7 pg/ml, respectively) than patients not administered DXM therapy (85.0 ± 152.1 pg/ml and 47.3 ± 287.6 pg/ml, respectively).

## Discussion

To the best of our knowledge, this study is the first attempt to demonstrate that DXM was effective in preventing CIA in mice, as evidenced by a decrease in paw swelling and significant reduction in histological synovitis in mice administered an intradermal injection of DXM. We also demonstrated that 6-month add-on DXM therapy significantly reduced the serum levels of proinflammatory cytokines including TNF-α, IFN-γ, and IL-17A in patients with RA. The therapeutic benefit of DXM in CIA might be related to its anti-inflammatory and immunomodulatory effects. The anti-inflammatory effects of add-on DXM therapy may be mediated through the inhibition of proinflammatory cytokine expression. Its immunomodulatory activity was revealed through the suppression of autoantibody production, as well as Th1 and Th17 responses, providing more evidence of the anti-inflammatory effects of DXM. The effectiveness of DXM in the inhibition of mouse CIA and the reduction of disease activity in human RA suggests that DXM may be a potential anti-arthritic agent with novel mechanisms of action.

Abundant evidence has demonstrated that proinflammatory cytokines play an important role in RA pathogenesis^1–5^. The overproduction of proinflammatory cytokines such as TNF-α and IL-6 is essential in the regulation of synovial inflammation and progression of RA^1–3^. In addition, the enhanced expression of IL-17A has been observed in rheumatoid synovium and synovial fluids of patients with early RA^24^. IL-17A is able to promote inflammation by enhancing the production of cytokines such as TNF-α, IL-1β, and IL-6^25^. Furthermore, injection of IL-17A is able to promote inflammation by enhancing the production of cytokines such as TNF-α, IL-1β, and IL-6 (Mills, 2008]. Furthermore, injection of IL-17A in a normal knee is sufficient to induce joint inflammation and bone and cartilage destruction^5^. Thus, therapeutic agents that alter proinflammatory cytokine profiles can be promising drugs in the treatment of RA^6–10^. Similar to a previous finding, our results showed that the oral administration of DXM caused a dose-dependent decrease in the expression of TNF-α, IL-6 and IL-17 in arthritic paw tissues and in the sera of CIA mice and patients with RA. Importantly, these data are considered evidence that DXM has an anti-inflammatory effect, which is effective against experimental arthritis in a mouse model and human RA patients in the present study.

Despite the inflammatory cytokines could contribute to the severity of RA, other therapeutic strategies, such as induction of immune tolerance, can be utilized to alleviate the RA in addition to suppression of inflammatory. A couple reports have demonstrated that the CD8 + regulatory T cells could be induced by type II collagen injected into the anterior chamber (AC) of the eye in CIA mouse model. By which, the increased immune tolerance prevented the further inflammatory immune responses either in CIA mouse model or DBA/1 mouse^26, 27^. Moreover, the reduction of reactive oxygen species (ROS) with free radical scavengers (e.g. phloretin) or other small molecule drugs (phosphodiesterase 4 inhibitors) can also be considered as an alternative therapy to treat autoimmune arthritis^28, 29^.

Although the exact immunomodulatory mechanism of DXM in CIA or RA patients remains unclear, we hypothesize that the efficacy of DXM treatment in CIA might be partially related to its blocking effect on DC functions and subsequent suppression of Th1 response, Th17 response, or both. Our hypothesis is supported by our previous findings includethe following: (1) DXM inhibited LPS-induced mouse and human DC maturation and blocked Ag (OVA)-specific T-cell proliferation and IFN-γ production^21^, and (2) DXM did not affect Th1 and Th17 differentiation in polarized-Th1 and Th17 differentiation conditions *in vitro* (Fig. 6), confirming that DXM interferes with DC function.

As an antitussive, the usual clinical dose of DXM in adult humans is 60–120 mg/day, and the estimated peak serum concentration is 8–16 μM^30^. The dose used in mice is 20 mg/kg, which is equivalent to a dose of 100 mg/60 kg in an adult human and is considerably lower than the toxic concentration (LD50 in rats = 350 mg/kg; IPCS INCHEM Database)^31^. Importantly, the present study demonstrated that DXM, which was orally administered at 20 mg/kg daily, ameliorated CII-induced arthritis and significantly inhibited joint inflammation and CII-specific immune responses. In addition, our *in vitro* study showed that 12.5 uM of DXM could inhibit DC maturation. Although a decrease in disease activity parameters (DAS28 score and CRP levels) was found in patients with RA administered and not administered DXM add-on therapy, a significant reduction in proinflammatory cytokines including TNF-α, IFN-γ, and IL-17A was observed only in those with DXM therapy. Overall, these results suggest that oral DXM treatment at a clinical antitussive dosage can decrease inflammation in CIA mice and patients with RA.

Although our study provides critical evidences toward to the potential therapeutic benefits and mechanism of action of DXM treatment, there are some limitations in this study that need to be addressed. First, this is a pioneer study that enrolled a small population of patients with active RA who had 24 weeks of follow-up. There was no significant change in the frequency of EULAR therapeutic responses in patients receiving 24-week add-on DXM treatment. It is possible that the duration of DXM treatment was not long enough to observe its effectiveness using EULAR response criteria. Second, the abnormal activation of macrophages and osteoclasts could play a role in the pathogenesis of autoimmune arthritis^32^, and DXM might therefore block the activation of macrophages and formation of osteoclasts *in vitro* and *in vivo* ^16, 33^.As a result, it is necessary to investigate the effects of DXM on the activation or differentiation of macrophages and osteoclasts in CIA mice or RA patients. Lastly, although we did not measure the production of granulocyte macrophage colony stimulating factor (GM-CSF), it cannot be neglected that the possibility of GM-CSF could modulate the dendritic cell differentiation as well as promote the population of regulatory T cells, which in turn alleviate the arthritis in CIA model^34–36^. Since there was no significant change in the numbers of FOXP3+ CD4+ regulatory T cells in our study, we anticipate the dual administration of DXM and GM-CSF might lead to a synergistic effect to achieve a better therapeutic outcome. In summary, our results showed that DXM has a potent effect against the development of mouse CIA and promotes a significant reduction in disease activity in human RA. The therapeutic effect of DXM could be related to the inhibition of proinflammatory cytokines and modulation of immune responses. In consideration of the cost-effectiveness and safety profile of DXM in treating RA, add-on DXM treatment could be a potential alternative therapeutic strategy. However, the use of DXM as an adjunct treatment in combination with traditional DMARDs in RA requires further study.

## Methods

### Animal study

#### Ethic statement

All animal procedures were conducted according to institutional guidelines and approved by the Institutional Animal Care and Utilization Committee of National Chung Hsing University, Taiwan (approval protocol no. NCHU-IACUC-104-027).

### Mice

Male DBA/1 mice (6–8-week-old) were obtained from the Jackson Laboratory, and kept under specific pathogen free conditions.

### CIA induction and DXM treatment

CIA was performed according to a previously described method^23^. Briefly, chick collagen type II (CII) (Chondrex, Inc) was dissolved in 10 mM of acetic acid to a 2-mg/ml concentration and emulsified with a complete Freund’s adjuvant (CFA, Sigma-Aldrich). At the beginning of the experiments (day 0), the mice were immunized with a 0.2-ml emulsion containing 100 ug of collagen at the tail base and were administered a booster (on day 21) with the same preparation of collagen and incomplete Freund’s adjuvant (IFA, Sigma-Aldrich). DXM (Sigma-Aldrich) was dissolved and freshly diluted in PBS. The mice were randomly divided into three groups (n = 5 per group). PBS (control group) and DXM (0.2 or 0.4 mg/kg) were orally administered once daily from day 21 to day 42 after the first immunization.

### Assessment of arthritis in a murine model

The severity of arthritis was measured in a double-blind manner using a semi-quantitative scoring system as previously described^37^. Each limb was scored using a scale from 0 to 4 based on increasing levels of erythema and swelling. The maximal arthritis score for each limb was 4, with 0 representing no swelling. The score for each mouse was the sum of the scores for the four limbs (maximum score = 16).

### Histopathological analysis

On day 42, the mice were killed, and representative ankle joints were collected, fixed with 10% buffered formalin, decalcified in 5% formic acid and embedded in paraffin. Each section (5 μm) of joint was stained with hematoxylin and eosin (H&E) for microscopic evaluation according to a previous study^38^. Histopathological changes in synovial hyperplasia and cell infiltration parameters were scored by a pathologist, and scores of 0–3 were based on the following criteria: 0, no changes; 1, mild changes; and 3, severe changes. Proteoglycan content in the articular cartilage was determined using Safranin O-fast green (Sigma-Aldrich) staining^39^. The loss of proteoglycan was scored using a scale from 0 to 3, ranging from fully stained cartilage to completely destroyed or unstained cartilage (with Safranin O) cartilage^40^.

### Determination of cytokine levels

The paws and sera were harvested from each group 42 days after the primary immunization. Protein extract was isolated from paw homogenate (100 mg of frozen tissue was homogenized in 1 ml of tissue lysis buffer with UltraCruz® Protease Inhibitor Cocktail, Santa Cruz Biotechnology). Protein concentrations were determined using a micro bicinchoninic acid assay (BCA; Pierce). Levels of proinflammatory cytokines including IFN-γ, TNF-α, IL-6, IL-10, and IL-17A were measured using ELISA following the manufacturer’s instructions (PeproTech).

### Analysis of anti-CII IgG antibody production

Serum samples were collected on day 42 post-immunization, and the anti-CII IgG, IgG2a, and IgG1 titers were measured using ELISA. Briefly, ELISA microtiter plates (Thermo Fisher Scientific) were coated with CII (10 μg/ml in PBS) at 4 °C overnight, followed by a blocking step with 3% bovine serum albumin (BSA) in Tris buffer for 1 h at room temperature. Tested sera were then serially diluted in Tris-buffered saline (pH, 8.0) containing 1% BSA and 0.5% tween-20 and added to a well at 4 °C overnight. After washing for five to seven times, bounded IgG was detected using an HRP-conjugated sheep anti-mouse IgG (Jackson Immunoresearch Laboratories) diluted to 1:5000, goat anti-mouse IgG2a diluted to 1:500, and goat anti-mouse IgG1 diluted to 1:500 as secondary antibodies (Abs). After washing, the plate was developed using ABTS (Roche Diagnostic Systems) as a substrate, and the reaction was stopped with H_2_SO_4_. The optical density (OD) was determined at 450 nm with an ELISA reader (Tecan Sunrise).

### Flow cytometry analysis

The inguinal lymph nodes (LNs) from each treated group were prepared. Briefly, on experimental day 42, inguinal LN cells were extracted from each mouse using mechanical disruption with mesh, and single-cell suspensions were obtained. For intracellular detection of cytokines, LN cells (2 × 10^6^) were stimulated with CII (20 ug/ml) for 72 h; Golgi stop solution (BD Biosciences) was added for 4 h before the culture cells were harvested. The cells were then washed twice in FACScan buffer and stained with a phycoerythrin (PE)-conjugated anti-mouse CD4 (Biolegend) Ab. Cells were then fixed and processed for intracellular staining using the Cytofix/Cytoperm Plus Kit (BD Biosciences) according to the manufacturer’s instructions. The FITC-conjugated mAbs specific to murine IFN-γ, IL-17A, and Foxp3 were purchased from BioLegend. All samples were detected using an Accuri 5 flow cytometer, and the mean fluorescence intensity was calculated using a C6 Accuri system software (Accuri Cytometer)

### Effect of DXM on the ability of DC to stimulate CII-specific T-cell response

The mouse BMDCs were generated as previously described^21^. Because our study showed that 12.5 μM of DXM could decrease LPS-induced BMDC function^21^, we used 6.25 and 12.5 μM of DXM as the initial concentrations for the following *in vitro* experiments. BMDCs were pretreated with DXM for 3 h. After incubation, the cells were harvested and washed with PBS. CD4^+^ T cells were positively enriched from the inguinal LNs of CIA mice (day 28) using MACS cell separation according to the manufacturer’s instruction (Miltenyi Biotec) and cultured with DXM-treated DCs (at DC:T cell = 1:5 ratio) in a 200-ul total volume per condition. In all experiments, in a 96-well flat-bottom plate (Corning), CII (20 ug/ml) or plate-bound anti-CD3 Abs (1 μg/well, Biolegend) were added to the co-culture wells and incubated for 96 h. Supernatants were harvested for measurement of cytokine production, and cell proliferation was detected using [^3^H] thymidine incorporation with a beta-counter (Beckman Instruments).

### Th1 and Th17 cell expansion *in vitro*

CD4^+^ T cells were purified from spleens of naïve DBA/1 mice by positive selection using microbeads against CD4 (Miltenyi Biotec) following the protocol provided by the manufacturer. Then, the purified naïve CD4^+^ T cells were seeded at 2 × 10^6^ per well into 96-well U-bottom microplates with RPMI 1640 medium (Hyclone) supplemented with 10% fetal bovine serum (Hyclone, Carlsbad, CA), 100 units/ml penicillin, and 100 μg of streptomycin (all from Invitrogen-Gibco). Th1 differentiation was driven by the of naïve CD4^+^ T cells with 1 μg/ml plate-bound anti-CD3 (clone 2C11, eBioscience), 1 μg/ml soluble anti-CD28 (clone PV1.17, eBioscience), 10 ng/ml IL-12 (PeproTech), 5 ng/ml IL-2 (PeproTech), and 2 μg/ml anti-IL-4 antibody (clone 11B11, eBioscience). Th17 differentiation was driven by the stimulation of naïve CD4^+^ T cells with 1 μg/ml plate-bound anti-CD3, 1 μg/ml soluble anti-CD28, 50 ng/ml IL-6 (PeproTech), 10 ng/ml TGF-β1 (PeproTech), 2 μg/ml anti-IFN-γ antibody (clone R4-6A2, eBioscience) and 2 μg/ml anti-IL-4 antibody. DXM was used at the dose indicated at the beginning of the Induction. Cells were harvested on day 4 of DXM treatment and analyzed for intracellular cytokines while culture supernatants were examined for cytokine levels by ELISA assay.

### RA patients study

#### Ethic statement

The institutional review board of Taichung Veterans General Hospital approved this study (C09060-1), and informed consent was obtained from all participants according to the Declaration of Helsinki. This trial is registered on ClinicalTrials.gov (NCT02368093) on 3/22/2015. All experiments were performed in accordance with relevant guidelines and regulations.

### Patients with RA

A total of 48 consecutive and biologic-naïve patients who fulfilled the 2010 criteria of the American College of Rheumatology (ACR) for RA^41^ were enrolled. Patients who had other concomitant autoimmune diseases, a history of allergy to DXM, or renal dysfunction (serum creatinine of >2.5 mg/dl) were excluded. All patients were randomized to a 6-month treatment with either oral DXM (dextromethorphan hydrobromide; Detosiv Slow Release^®^ [60 mg per tablet, Lotus Pharmaceutical Company, Taipei, Taiwan] with *T*_1/2_ = 7.75 h; *T*_max_ = 4.83 h; *C*_max_ = 14.6 ng ml–1; and mean residual time = 5.86 h; 120 mg per day with a once-daily dose taken after breakfast) or placebo pills with an appearance similar to that of the DXM tablets. Randomization was performed by the pharmacy at the Taichung Veterans General Hospital. Non-studied medications were not changed during the course of study.

Twenty-four patients received add-on DXM therapy, and the other 24 patients received traditional DMARDs alone in a stable dose. DMARDs including methotrexate, sulfasalazine, hydroxychloroquine, leflunomide, and cyclosporine were used. Disease activity was assessed using a 28-joint disease activity score (DAS28)^42^ before starting the add-on DXM therapy (as a baseline) and at the end of the 6-month therapy with or without add-on DXM. DAS28 was evaluated by one rheumatologist who did not know the medications used or laboratory test results. Patients were categorized as good, moderate, or poor responders based on the amount of change in the DAS28 and DAS28 level reached. At the evaluation time, good responders were defined as those with a decrease in DAS28 from baseline (∆DAS28) of >1.2 and a DAS28 of ≤3.2; moderate responders had either ∆DAS28 of >1.2 and a DAS28 of >3.2 or ∆DAS28 of 0.6–1.2 and a DAS28 of ≤5.1; and poor responders had either ∆DAS28 of <0.6 or a DAS28 of >5.1.

### Determination of serum C-reactive protein (CRP), rheumatoid factor (RF)-IgM, and anti-cyclic citrullinated peptide (CCP) Ab levels

The serum CRP and RF-IgM levels were measured with a nephelometry (Dade Behring Inc). Determination of anti-CCP Abs was performed using an ELISA commercial kit (INOVA Diagnostics Inc). Patients with positive results for RF-IgM or anti-CCP Abs were considered seropositive.

### Statistical analysis

The results are presented as mean ± standard deviation (SD), standard error of the mean (SEM), or median (interquartile range). For both *in vitro* and *in vivo* studies, statistical analysis was performed using one-way ANOVA (ANalysis Of VAriance) with a post-hoc Tukey HSD (Honest Significant Differences) test to compare multiple treatments using GraphPad Prism (version 5 for Windows; GraphPad Software). The nonparametric Kruskal–Wallis test was used for comparison between groups in clinical study. The independent sample *t* test was used for comparison between groups in EULAR therapeutic response and cytokine levels. The correlation coefficient was calculated using the nonparametric Spearman’s rank correlation test. The Wilcoxon signed rank test was employed to compare disease activity parameters including DAS28, CRP levels, and cytokine levels during the follow-up of patients with RA at baseline and after 6-month therapy with or without add-on DXM. It was considered significant if the result was < 0.05.
